# 

**Published:** 1972-10

**Authors:** 


					BRISTOL
MEDICO
CHIRURGICAL
JOURNAL
?CTOBER 1972
VOL. 87 (iv) No. 324

				

